# Allethrin Promotes Apoptosis and Autophagy Associated with the Oxidative Stress-Related PI3K/AKT/mTOR Signaling Pathway in Developing Rat Ovaries

**DOI:** 10.3390/ijms23126397

**Published:** 2022-06-07

**Authors:** Maroua Jalouli, Afoua Mofti, Yasser A. Elnakady, Saber Nahdi, Anouar Feriani, Abdelkarem Alrezaki, Khaled Sebei, Mariano Bizzarri, Saleh Alwasel, Abdel Halim Harrath

**Affiliations:** 1 Department of Zoology, College of Science, King Saud University, Riyadh 11451, Saudi Arabia; maroua.jalouli@gmail.com (M.J.); yelnakady@ksu.edu.sa (Y.A.E.); nehdisabeur@gmail.com (S.N.); aayr1978@gmail.com (A.A.); salwasel@ksu.edu.sa (S.A.); 2 Laboratory Microorganismes and Active Biomolecules, Faculty of Sciences of Tunis, University of Tunis El Manar, Tunis 1068, Tunisia; 3 Laboratory of Biotechnology and Biomonitoring of the Environment and Oasis Ecosystems, University of Gafsa, Gafsa 2112, Tunisia; aoufamoufti@gmail.com (A.M.); ferianianwer@yahoo.fr (A.F.); 4 Protéomie Fonctionnelle & Potentiel Nutraceutique de la Biodiversité de Tunisie (UR13/ES39), ISSBAT, University of Tunis El Manar, Tunis 1068, Tunisia; khaled.sebei@issbat.utm.tn; 5 Department of Experimental Medicine, Sapienza University of Rome, Viale Regina Elena 324, 00161 Rome, Italy; mariano.bizzarri@uniroma1.it

**Keywords:** female infertility, pyrethroid insecticide, allethrin, reproductive toxicity, oxidative stress, autophagy, PI3K/AKT, apoptosis

## Abstract

The increased concern regarding the reduction in female fertility and the impressive numbers of women undergoing fertility treatment support the existence of environmental factors beyond inappropriate programming of developing ovaries. Among these factors are pyrethroids, which are currently some of the most commonly used pesticides worldwide. The present study was performed to investigate the developmental effects of the pyrethroid-based insecticide allethrin on ovarian function in rat offspring in adulthood. We mainly focused on the roles of oxidative stress, apoptosis, autophagy and the related pathways in ovarian injury. Thirty-day-old Wistar albino female rats were intragastrically administered 0 (control), 34.2 or 68.5 mg/kg body weight allethrin after breeding from Day 6 of pregnancy until delivery. We found that allethrin-induced ovarian histopathological damage was accompanied by elevations in oxidative stress and apoptosis. Interestingly, the number of autophagosomes in allethrin-treated ovaries was higher, and this increase was correlated with the upregulated expression of genes and proteins related to the autophagic marker LC-3. Furthermore, allethrin downregulated the expression of PI3K, AKT and mTOR in allethrin-treated ovaries compared with control ovaries. Taken together, the findings of this study suggest that exposure to the pyrethroid-based insecticide allethrin adversely affects both the follicle structure and function in rat offspring during adulthood. Specifically, allethrin can induce excessive oxidative stress and defective autophagy-related apoptosis, probably through inactivation of the PI3K/AKT/mTOR signaling pathway, and these effects may contribute to ovarian dysfunction and impaired fertility in female offspring.

## 1. Introduction

An individual’s health and chances for survival in later life depend on fetal growth [1], which is related to the “Barker hypothesis” of the developmental origin of adult diseases [2]. In experimental animals and humans, low birth weight is an indicator of nonoptimal prenatal development. Any environmental disturbances can have a harmful effect on fetal life, which subsequently leads to permanent diseases [3]. For example, a fetus whose mother has been exposed to a variety of environmental compounds may undergo physiological adaptation in response to changes in the embryonic environment to prepare for life after birth in so-called fetal programming [4,5]. Humans are often exposed to chemical compounds, particularly synthetic endocrine-disrupting chemicals (EDCs) that contaminate our environment and our food sources; such chemicals may change the embryonic environment, leading to inappropriate programming of developing organ systems [6]. The increased prevalence of some common diseases, such as female infertility, could be related to the developmental exposure of some female reproductive organs, mainly the ovaries, to environmental pollutants that can adversely influence the developmental trajectory of target tissue differentiation. Currently, impressive numbers of women are undergoing fertility treatment [7], and there is increasing evidence showing that environmental factors may at least partially explain these high numbers and have implications that go beyond “natural” female infertility.

Among the environmental factors that are currently raising major concerns are pesticides used to boost agricultural production, which leak into water sources, the environmental landscape and the food chain and thus pose a danger to all forms of life [8]. Particularly problematic are pyrethroid insecticides, which comprise a major class of insecticides that are synthetic analogs of the naturally occurring insecticide pyrethrin found in the flowers of *Chrysanthemum cineraraefolium* [9]. Pyrethroids have gained popularity because of their low association with acute toxic effects and their high effectiveness against insects. Recent studies have reported the widespread presence of pyrethroid pesticides in food products as well as widespread exposure among the general population in the United States and worldwide [10]. Metabolites of pyrethroids have been detected even in breast milk and urine [8,11]. The extensive usage of pyrethroids has attracted public concern, particularly because these insecticides are considered EDCs [12].

Previous investigations on the effects of pyrethroid exposure on female ovarian functions have shown that pyrethroids inhibit estradiol and progesterone production [13] and impair the follicular and corpus luteal cell morphology [14]. Pyrethroid exposure can also affect female fertility by decreasing the levels of the ovarian reserve predictor anti-Mullerian hormone (AMH), which leads to diminished ovarian reserve [12,15]. Although in vitro and animal studies have demonstrated that exposure to pyrethroids may affect ovarian function, the available evidence of the consequences of chronic exposure to these insecticides on long-term female reproductive health outcomes in animals and humans remains limited. A very recent study using mice provided the first demonstration of the effect of gestational exposure to pyrethroids on the ovaries of female offspring in adulthood [16]. That study showed irreversible adverse effects on the fertility of mouse offspring, including a significant decrease in the number of primary follicles and significant increases in the number of atretic follicles and granulosa cell apoptosis [16]. However, the mentioned study used a mixture of eight pyrethroids (fenvalerate, λ-cyhalothrin, fenpropathrin, deltamethrin, cyfluthrin, cypermethrin, permethrin and bifenthrin) that did not include allethrin, which is one of the most commonly used pyrethroids. Exposure to the pyrethroid-based pesticide allethrin may be a major problem for human health and may notably affect ovarian structure and function, as it has been detected in several commonly consumed foods, such as cereals and vegetables [17]. Allethrin is considered a synthetic chemical that is harmful to living organisms because it is a membrane-active substance that affects membrane phospholipids and thus causes tissue damage [18,19]. To our knowledge, the current study comprises the first investigation of the effects of in utero exposure to the pyrethroid-based insecticide allethrin on ovarian function in adulthood in mammals. In the study, female Wistar albino rats were exposed daily after breeding to allethrin at environmentally relevant concentrations to mimic human exposure occurring during the critical perinatal phase. We found that developmental exposure to allethrin induces excessive oxidative stress, apoptosis and autophagic processes, probably through inactivation of the PI3K/AKT/mTOR signaling pathway, and these effects contribute to disrupted ovarian function in female offspring during adulthood.

## 2. Results

### 2.1. Allethrin Caused Histopathological Damage in Rat Ovaries

**Figure 1:**
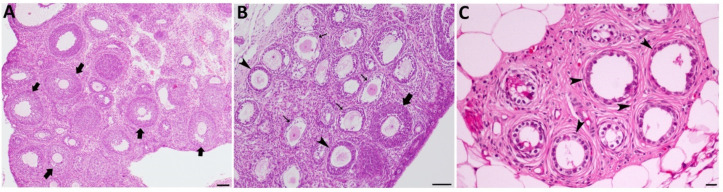
Allethrin exposure exerted toxic effects on ovarian function. Photomicrographs of ovarian sections from the control group (**A**) showed normal ovarian structures containing normal growing follicles (large arrows), whereas photomicrographs of allethrin-treated ovaries (**B**) showed altered structures in most of the growing follicles (arrows), as revealed by oocyte vacuolization and degradation, and this finding was obtained with both doses. Although few normal follicles were observed (large arrows), cystic dilation of follicles with decreased numbers of sparse granulosa cells was also observed with both exposure doses (**B**,**C**). Some of the ovaries from the higher dosage treated groups were reduced in size and contained cysts and degenerating follicles (**C**).

Compared with ovaries from the control group, which had normal structures (Figure 1A), ovaries from the groups treated with low and high doses of allethrin showed altered structures in most of the growing follicles. Specifically, cystic dilation of follicles and decreased numbers of sparse granulosa cells were observed after exposure to both doses (Figure 1B,C). Some of the ovaries from the treated groups were reduced in size and contained cysts and degenerating follicles (Figure 1C).

### 2.2. Allethrin Induced Oxidative Stress in Rat Ovaries

**Figure 2:**
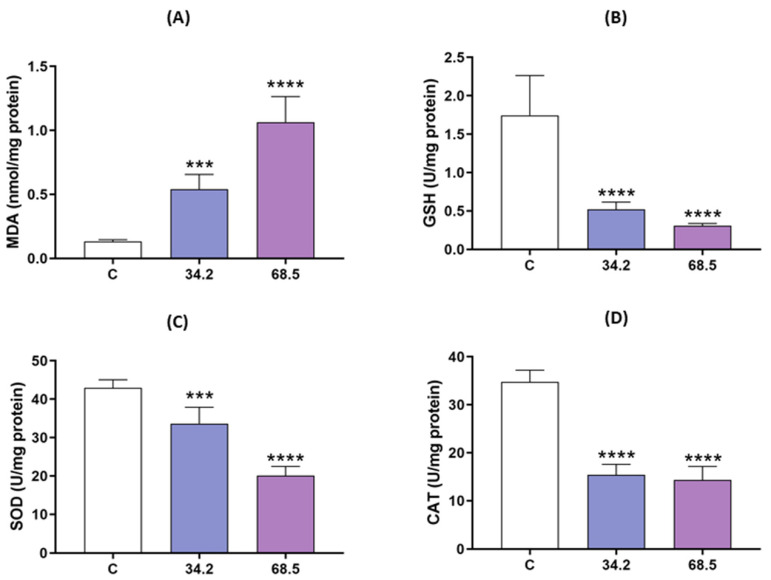
Allethrin treatment-induced oxidative stress. Pregnant female rats were exposed to 34.2 and 68.5 mg of allethrin/kg body weight from Day 6 of gestation until delivery, and the levels of MDA (**A**), GSH (**B**), SOD (**C**) and CAT (**D**) in female offspring were then determined during the prepubertal stage. All data are expressed as the means ± SDs. *** *p* < 0.0005; **** *p* < 0.00001.

To detect the involvement of oxidative stress in allethrin-induced damage in rat ovarian cells, the levels of MDA as well as the antioxidant enzyme activity of GSH, SOD and CAT were measured. As shown in Figure 2, the MDA content was increased in a dose-dependent manner in the allethrin-treated ovarian cells compared with the control cells (*p* < 0.05) (Figure 2A), whereas the GSH content and the activity of SOD and CAT were significantly decreased (*p* < 0.0005) (Figure 2B–D). These results indicated that allethrin induced oxidative stress in rat ovarian cells.

### 2.3. Allethrin Induced Autophagic and Apoptotic Marker Expression

**Figure 3:**
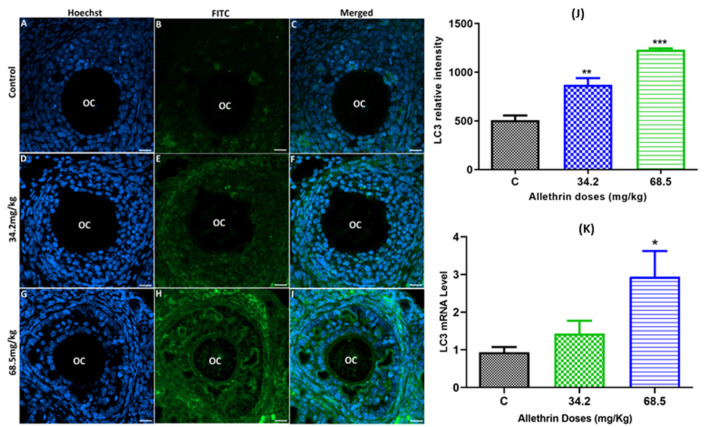
Autophagy in ovarian tissue was evaluated by immunofluorescence staining (**A**–**I**) and assessment of the relative fluorescence intensity (**J**) of LC-3 in the control and exposed groups. Additionally, the mRNA levels of the LC-3 gene in female rats exposed to allethrin compared with those in the control rats were determined by RT–PCR (**K**). Scale bar = 200 μm. * *p* < 0.05; ** *p* < 0.005; *** *p* < 0.0001.

To determine whether autophagy is involved in allethrin-induced ovarian toxicity, the autophagic marker LC3 was immunolocalized, and the expression of its corresponding gene was evaluated by RT–PCR. We found that allethrin significantly increased the LC3-related fluorescence intensity in a dose-dependent manner compared with the control level (Figure 3A–J). Moreover, allethrin exposure increased the mRNA level of the LC3 gene (Figure 3K), but a quantitative analysis confirmed that a significant increase was obtained only with the high dose (68.5 mg/kg), and no significant effect was observed with the dose of 34.2 mg/kg.

**Figure 4:**
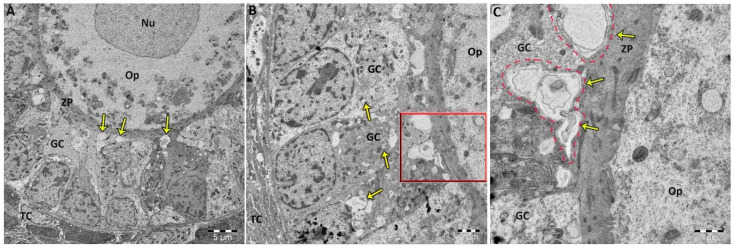
Transmission electron micrographs revealed significant increases in the numbers of autophagic vacuoles (arrows) in granulosa cells from females treated with low (**A**) and high doses of allethrin (**B**,**C**). Autophagic vacuoles are double-membrane structures that contain a number of atypical and/or injured mitochondria, endoplasmic reticulum membranes and cytoplasm (**C**). However, these autophagosomes were not detected in oocytes or theca cells. GC: granulosa cell, Nu: nucleus, Op: ooplasm, TC: theca cell and ZP: zona pellucida.

TEM examination of allethrin-exposed ovaries indicated the presence of normally structured oocytes containing typical cytoplasmic organelles, including mitochondria and an endoplasmic reticulum (Figure 4). However, the granulosa cells of allethrin-exposed ovaries exhibited large numbers of typical autophagosomes, indicating the occurrence of autophagy (Figure 4A–C). The high-resolution transmission electron micrographs revealed significantly higher numbers of autophagic vacuoles in the high-dose groups (B and C) compared with the low-dose group (A).

**Figure 5:**
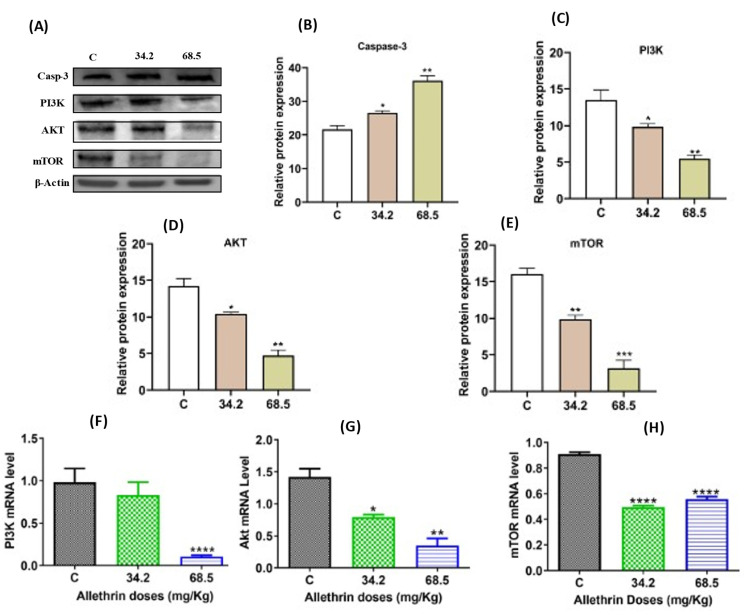
Effects of allethrin on the expression of apoptosis and autophagy markers at the protein and mRNA levels in the rat ovaries (**A**–**E**). The protein expression levels of active caspase-3, PI3K, AKT and mTOR were examined by western blotting. The mRNA expression levels of the genes encoding PI3K, AKT and mTOR were detected by RT–PCR (**F**–**H**). All data are expressed as the means ± SDs. * *p* < 0.05; ** *p* < 0.005; *** *p* < 0.0001; **** *p* < 0.00001.

Western blot analysis showed that the proapoptotic marker active caspase-3 was significantly increased in the groups treated with the low (*p* < 0.05) and high doses (*p* < 0.005) of allethrin compared with the control group (Figure 5A,B).

### 2.4. Allethrin Inhibited the PI3K/AKT/mTOR Signaling Pathway

To elucidate the mechanisms that mediate the effects of allethrin on ovarian cells, the PI3K/AKT/mTOR signaling pathway was analyzed. Western blot analysis showed that PI3K and AKT protein expression levels were significantly decreased in a dose-dependent manner in the allethrin-exposed groups compared with the control group (Figure 5A,C,D) (*p* < 0.01). Similarly, the mTOR protein levels were significantly decreased in both treated groups (Figure 5A,E) (*p* < 0.005). Consistent with the protein expression levels, a significant decrease in the mRNA level of the PI3K gene was observed in the group treated with the high dose of 68.5 mg/kg (Figure 5F), whereas the mRNA levels of the AKT and mTOR (Figure 5G,H) genes were significantly decreased in all allethrin-treated groups compared with the control group. Collectively, these results indicated that the PI3K/AKT/mTOR pathway might mediate the effect of allethrin on ovarian cell function.

## 3. Discussion

Allethrin is regarded as a health threat because it can affect various organs of the human body, such as the nervous system [20,21], the cardiovascular system [21,22], the liver [20] and even the male reproductive system [23,24]. However, the toxic effect of allethrin on the female reproductive system and the underlying mechanism remain unknown. In the present study, female rats were orally exposed to 34.2 and 68.5 mg/kg allethrin. We found that allethrin significantly affected the structure and function of ovarian tissue, which indicated that allethrin exposure might pose a high risk of damage to the female reproductive system.

It is well known that reactive oxygen species (ROS) are produced in the ovaries as byproducts of normal physiological metabolism, and antioxidants work to maintain the balance between ROS production and excretion to maximize cell efficiency. However, excessive ROS production causes an imbalance between oxidation and antioxidation, leading to oxidative stress. Oxidative stress, the principal factor affecting oocyte quality, leads to oocyte aging and a decline in fertility [25]. We examined the relationship between oxidative stress and ovarian toxicity through four established parameters: the contents of MDA and GSH and the activities of SOD and CAT. Our results showed that allethrin treatment generally increased the levels of MDA in ovarian tissue and decreased the levels of GSH and the enzyme activities of SOD and CAT. This imbalance between oxidative/antioxidative processes may suggest that oxidative stress is a major mechanism of allethrin-induced ovarian toxicity that causes some pathological consequences and perhaps many problems during oogenesis and folliculogenesis [26], as shown by our histopathological results. Indeed, some female reproductive diseases, such as endometriosis and PCOS, occur when there is an imbalance between ROS production and antioxidants [27]. Moreover, recent studies have suggested that oxidative stress contributes significantly to reproductive toxicity [28,29]. Liu et al. indicated that nonylphenol can induce oxidative stress, which plays an important role in the apoptosis of rat ovarian granulosa cells [30]. In addition, polycyclic aromatic compounds can affect ovarian follicle development via increased production of ROS [31,32]. Notably, the exposure of mice to benzo(a)pyrene and dimethylbenz[a]anthracene significantly increases the ROS levels, leading to increased granulosa and theca cell apoptosis [32,33]. These results are consistent with our results because we found that allethrin induced oxidative stress. The induced oxidative stress may have led to ovarian cell apoptosis because we found upregulated expression of the executor of apoptosis, active caspase 3. Proliferation and cell death by apoptosis are continuous processes in mammalian ovaries [34], and apoptosis may enable the elimination of poor-quality oocytes in prepubertal mouse ovaries [35]; these mechanisms may explain the detection of active caspase-3 in control ovaries.

Autophagy is a highly conserved self-renewal process that can remove dysfunctional proteins and organelles [36]. An increase in autophagy that occurs as an adaptation to stress can lead to reduced apoptotic cytotoxicity, whereas abnormal/excessive autophagy can accelerate apoptotic cell death [37,38,39]. A large number of studies have found that oxidative stress can induce apoptosis, autophagy, or both, leading to harmful effects on reproductive function [40,41]. In agreement with these results, the findings of the current study revealed the appearance of large numbers of autophagosomes, the gold-standard markers of autophagy, in the allethrin-treated ovaries [42]. In correlation with the TEM results, the protein and mRNA levels of LC3 were significantly higher in the allethrin-treated ovaries than in the control ovaries. This enhanced autophagy was accompanied by an increased MDA level and reduced SOD, GSH and CAT levels, which suggested that oxidative stress induced autophagy in the allethrin-treated ovaries. ROS reportedly activate autophagy in follicular granulosa cells via the mTOR pathway, indicating a close correlation between oxidative stress and autophagy [43]. In addition, consistent with our results, the inactivation of ROS generation has been found to inhibit both apoptosis and autophagy in Sertoli cells [44]. This activation of autophagy is an important first step for ensuring the balance between ROS and oxidant scavengers to protect cell viability, but in the case of excessive ROS production, such as that occurring in our study, cell death can occur as a result of autophagy failure to repair cells [43]. This finding may explain the increased levels of the proapoptotic marker caspase-3 detected in allethrin-treated ovaries because autophagy can be a mechanism of caspase- and apoptosis-independent cell death [45].

In mammalian cells, the PI3K/AKT/mTOR pathway plays critical roles in many cellular activities, such as cell survival, cell proliferation and growth [46,47]. It is also an important intracellular signaling pathway that regulates autophagy [48]. Our results showed that the mRNA levels of the PI3K, AKT, AMPK and mTOR genes were significantly lower in allethrin-treated ovaries than in normal ovaries. Similarly, according to the western blot data, the protein levels of PI3K, AKT and mTOR were significantly decreased. These findings suggest that inhibition of the PI3K/Akt/mTOR signaling pathway may play a role in allethrin-induced autophagy, and this process may be regulated by enhanced oxidative stress. In fact, because the mTOR levels were decreased in allethrin-treated ovaries, autophagy may have been induced, as shown by the appearance of autophagic vacuoles and the overexpression of LC3-related genes and proteins. It is widely acknowledged that mTOR, the downstream target of PI3K and AKT, is the core modulator of autophagy because its suppression leads to stimulation of autophagy [49]. In addition, the inhibition of ROS generation negatively affects apoptosis and autophagy [44], and ROS-mediated autophagy is associated with inactivation of the PI3K/AKT/mTOR signaling pathway [40,50]. Consistent with our results, a large number of toxicants induce oxidative stress-mediated autophagy by downregulating the PI3K/AKT/mTOR pathway or inhibiting mTOR [51]. Among these toxicants, honokiol induces autophagic cell death through ROS generation and inactivation of the PI3K/AKT/mTOR signaling pathway [52]. In addition, the toxicants nonylphenol and aflatoxin B1 cause male reproductive damage through induction of autophagy and downregulation of the PI3K/AKT/mTOR signaling pathway [53,54]. Similarly, Yang et al. [55] found that the treatment of mouse liver cells with increasing concentrations of aconitine increases the expression of autophagy marker proteins but significantly decreases the levels of the proteins p-PI3K, p-AKT, and p-mTOR.

## 4. Materials and Methods

### 4.1. Animal Treatment and Sampling

Thirty pubertal virgin female Wistar albino rats (200–250 g and 60 days of age) were kept individually in cages in a well-ventilated room with a temperature of 21 ± 1 °C, a humidity-controlled (60−80%) atmosphere and a 12 h light:12 h dark cycle. The rats were given distilled water and food ad libitum. After one week of acclimatization, the rats were weighed and randomly divided into one control group and two allethrin-treated groups; those in the two latter groups were treated by gavage (34.2 and 68.5 mg/kg BW starting from Day 6 of gestation, which corresponds to the implantation of the fetus, until delivery). Based on the reported oral LD50 values of allethrin (685 mg/kg) [56] and based on the results of previous studies on the effects of allethrin on different biological systems [57,58,59], we used the 1/10 and 1/20 oral LD50 values of allethrin, which correspond to 68.5 and 34.2 mg/kg BW, respectively. After parturition, we obtained the female offspring that were euthanized when they reach 28 days of age (prepuberty), and their ovaries were removed, cleaned and labeled according to their origin (groups). The ovaries were immediately fixed in neutral buffered formalin (NBF) for histopathological and immunofluorescence studies or fixed in RNAlater solution and stored at −80 °C for molecular biology assays (western blotting and RT–PCR). All animal procedures were conducted after approval was obtained from the Ethical Committee for the Care and Use of Laboratory Animals at the University of Gafsa, Tunisia.

### 4.2. Histological Preparation

The ovary samples fixed in 10% NBF were dehydrated in increasing concentrations of ethanol and embedded in paraffin blocks. For the histopathological study, the blocks were serially cut into 5-µm-thick sections using a rotary microtome and then stained with hematoxylin-eosin (H&E). Some blocks were cut into 3-µm-thick sections for immunofluorescence analysis.

### 4.3. Oxidative Stress Measurement

Ovarian tissues (3 per group) were homogenized with a glass homogenizer in precooled physiological saline. The cells in the prepared homogenized solution were lysed by ultrasonic cell disruption and then centrifuged at 13,000× *g* (4 °C) for 15 min to obtain the tissue supernatant. The supernatant was used to measure the levels of malondialdehyde (MDA) based on a thiobarbituric acid reaction using a spectrophotometer at an absorbance of 532 nm (Shimadzu UV-1800, Shimadzu, Canby, OR, USA) and expressed as nmol concentration of MDA content per mg of protein, as described previously by Buege and Aust [60]. The amount of GSH in the tissue homogenate was estimated as reported by Sedlak et al. [61] with a few modifications. A volume of 1.5 mL of supernatant was added to 1.5 mL of Tris-HCl buffer (200 mM), 0.5 mL of EDTA at pH 7.5 (0.2 mM), 0.1 mL of DTNB (10 mM) and 0.79 mL of methanol, and vortexed and incubated at 37 °C for 30 min. The absorbance was measured at 412 nm using a spectrophotometer (Shimadzu, UV-1800). The activity of catalase (CAT) was measured using the method described by Aebi [62] with slight modifications. Briefly, 1 mL of assay buffer (50 mM potassium phosphate buffer, pH 7.0) containing H_2_O_2_ (100 mM) was added to 20 µL of the ovarian homogenate. The optical density was calculated for 120 s at 240 nm. The results are expressed as units of catalase activity (mg protein). The total activity of SOD was evaluated by measuring the inhibition of pyrogallol autoxidation catalyzed by superoxide radicals based on the method described by Marklund and Marklund [63]. The absorbance values at 480 nm were recorded and expressed as nM mg protein. The protein levels of the ovarian homogenate were measured to estimate the activity per mg protein.

### 4.4. Western Blot Analysis

The homogenized ovarian tissues were lysed in RIPA lysis buffer containing a protease inhibitor. The supernatants obtained after centrifugation for 15 min at 13,000 rpm were then collected for western blotting. The protein concentration was detected by the Bradford test. Thereafter, the proteins were separated by Mini-PROTEAN^®^ TGX™ (Bio-Rad, Hercules, CA, USA) and transferred onto PVDF membranes using a Trans-Blot Turbo Transfer System. The PVDF membranes were blocked with 5% horse serum at room temperature for 3 h and incubated at 4 °C overnight with rabbit polyclonal antibodies against caspase-3 (diluted 1:2000; ab184787, Abcam, Cambridge, UK), total PI3K (1:1000, ab191606, Abcam, Cambridge, UK), total AKT (1:500, ab8805, Abcam, Cambridge, UK), and total mTOR (1:800, Dg-Peptide Co., Hangzhou, China). Subsequently, the primary antibodies were incubated with the corresponding secondary antibodies (1:5000 sc-2357 and sc-516102 Sigma-Aldrich, St. Louis, MO, USA) for 2 h at 37 °C. The bands of the target proteins were imaged using a Bio-Rad Gel Documentation System and analyzed using Image Lab software (Bio-Rad, USA).

### 4.5. Immunofluorescence Staining and Confocal Microscopy

Immunofluorescence analysis was performed according to the methods used in our previous studies [64,65]. Briefly, 3-µm-thick sections of ovarian tissues were dewaxed, hydrated with gradient ethanol and washed twice with distilled water and three times with 1x PBS. Thereafter, the sections were placed in 0.1% Triton X-100 with 0.1% sodium citrate, blocked with FBS and incubated overnight at 4 °C with an anti-LC-3 (1:100 dilutions, Dg-Peptide Co., Hangzhou, China) primary antibody. After washing with 1x PBS, the sections were incubated with Alexa Fluor 488 anti-rabbit (1:2000 dilutions, Abcam, Boston, MA, USA) for 45 min at room temperature (RT) in the dark. The sections were then washed with PBS and TE buffer before Hoechst solution was added (diluted 1:15,000, Hoechst 33342, Life Technologies, Waltham, MA, USA). The sections were observed and imaged for signal quantification with a spinning disk confocal microscope from Zeiss. The signal intensity was analyzed and used for the quantification of protein expression with the Zen 3.1 service (ZEN lite).

### 4.6. Ultrastructure Evaluation

For transmission electron microscopy (TEM), ovarian tissues were fixed overnight with 2.5% glutaraldehyde in 0.1 M phosphate buffer (pH 7.2) at 4 °C, postfixed in 1% OsO4, dehydrated for 1 h through an increasing ethanol series and embedded in Epon. Finally, the ultrathin sections were double-stained with 2% uranyl acetate and lead citrate, and electron micrographs were captured using a transmission electron microscope (JEOL JEM-1011, JEOL Ltd., MA, USA) and Gathan^TM^ software at the Research Center, King Saud University, Riyadh, Saudi Arabia.

### 4.7. Analysis of Gene Expression (RT–PCR)

Total RNA was isolated from ovarian tissues with a RNeasy Mini Kit (Qiagen, Westburg, The Netherlands) and was then reverse-transcribed into cDNA using an iScript™ cDNA synthesis kit (Applied Biosystem, Carlsbad, CA, USA) according to the manufacturer’s instructions. The quality and integrity of the extracted RNA were verified by measuring the 260/280 nm ratio using a NanoDrop. Real-time PCR (RT–PCR) was performed using SYBR Green, gene-specific primers (Table 1) and an Applied Biosystems 7500 Fast RT–PCR system (Carlsbad, CA, USA) with the following protocol: 1 cycle of initial denaturation at 95 °C for 2 min and 40 cycles of 94 °C for 20 s, 58 °C for 20 s and 72 °C for 20 s. For each gene transcript, the relative amount was calculated with the 2^−ΔΔCT^ method using GAPDH as the reference gene for normalization.

### 4.8. Statistics

The statistical significance of the differences in the mean values between the treated groups and the control group were analyzed by one-way ANOVA followed by Tukey’s multiple comparisons test using GraphPad Prism 9.3.0 (463) software. *p* < 0.05 was considered to indicate significance. All the data are presented as the means ± standard deviations (SDs).

## 5. Conclusions

**Figure 6:**
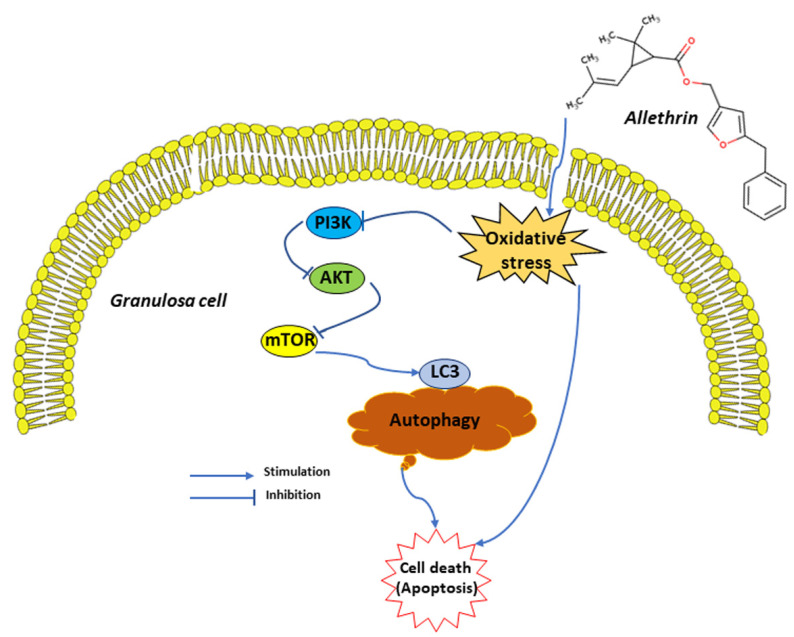
Schematic diagram of the effects of allethrin on ovarian cells, mainly granulosa cells.

In conclusion, the results reported herein provide evidence showing that allethrin treatment increases the levels of oxidative stress, apoptosis and autophagy in ovaries and downregulates the gene and protein expression of PI3K, AKT and mTOR (Figure 6). Many studies have reported that oxidative stress can induce apoptosis, autophagy or both [41].

Considering the numerous autophagosomes with enclosed mitochondria-like organelles and the elevated expression of autophagic markers in allethrin-treated ovaries compared with control ovaries, we postulate that autophagy might play an important role in the oxidative stress response. In fact, autophagy is induced to reduce oxidative stress and thus protect ovarian cells, as demonstrated by previous findings showing that autophagy inhibition increases the ovarian ROS levels [66]. In contrast, apoptosis and autophagy are interconnected and tightly regulated processes [55], and several studies have shown that autophagy can be a mechanism of caspase- and apoptosis-independent cell death [45]. We speculate that massive cell death via autophagy-related apoptosis may have occurred in the allethrin-treated ovaries due to the failure of autophagy and oxidant scavengers to repair ovarian cells, including theca and granulosa cells. This effect may have led to impaired female fertility and probably led to the development of a PCOS-like phenotype. However, more in vitro and in vivo studies are needed to obtain a more comprehensive understanding of the crosstalk among oxidative stress, autophagy and apoptosis and to elucidate how each process controls the others in the context of the in utero effects of allethrin on female fertility parameters.

## Figures and Tables

**Table 1:** Primers for real-time RT–PCR.

Gene Symbol	Sequences
*LC3*	F: TGTTAGGCTTGCTCTTTTGG R: GCAGAGGAAATGACCACAGAT
*PI3K*	F: GATGTCTGCGTTAGGGCTTACC R: TCAGCATCATGGAGAACAGGAT
*AKT*	F: CTCATTCCAGACCCACGAC R: ACAGCCCGAAGTCCGTTA
*mTOR*	F: TGCCTTCACAGATACCCAGTAC R: AGGTAGACCTTAAACTCGGAC
*GAPDH*	F: GCATCTTCTTGTGCAGTGCC R: GATGGTGATGGGTTTCCCGT

## Data Availability

The data that support the findings of this study are available from the corresponding author (Abdel Halim Harrath) upon reasonable request.
